# Clinical Outcomes and Predictors of Response to Levodopa–Entacapone–Carbidopa Intestinal Gel in Advanced Parkinson’s Disease: A Retrospective Cohort Study over a 4-Year Period

**DOI:** 10.3390/pharmaceutics18050517

**Published:** 2026-04-23

**Authors:** Károly Orbán-Kis, Róbert Máté Szász, Szabolcs Szatmári, Viorelia Adelina Constantin, Simona Maria Bățagă, János Szederjesi, Ilie-Marius Ciorba, Előd Ernő Nagy, Radu Mircea Neagoe, Krisztina Kelemen, Szabolcs Szatmári, Konrád-János Bíró, Nicoleta Crăciun Ciorba, Attila Frigy, József Attila Szász

**Affiliations:** 1 George Emil Palade University of Medicine, Pharmacy, Science and Technology of Târgu Mures, 540142 Târgu Mureș, Romania; karoly.orban-kis@umfst.ro (K.O.-K.); krisztina.kelemen@umfst.ro (K.K.); szaszneuro@yahoo.com (J.A.S.); 2 2nd Clinic of Neurology, Mures County Emergency Clinical Hospital, 540136 Târgu Mureș, Romania; vioreliaconstantin@yahoo.com (V.A.C.);; 3 Department of Gastroenterology, Mures County Emergency Clinical Hospital, 540136 Târgu Mureș, Romania; 4 Laboratory of Medical Analysis, Clinical County Hospital Mures, 540394 Târgu Mureș, Romania; 5 2nd Clinic of Surgery, Mures County Emergency Clinical Hospital, 540136 Târgu Mureș, Romania; 6 Department of Neurology, Semmelweis University, 1083 Budapest, Hungary; szatmari.szabolcs@semmelweis.hu; 7 Department of Internal Medicine IV, Clinical County Hospital Mures, 540136 Târgu Mureș, Romania

**Keywords:** advanced Parkinson’s disease, device-aided therapy, intrajejunal infusion, LECIG, motor fluctuations, dyskinesia, 5-2-1 criteria, tertiary care

## Abstract

**Background/Objectives**: Advanced Parkinson’s disease is characterized by severe motor fluctuations and disabling dyskinesias that often become refractory to conventional oral dopaminergic therapies. **Methods**: This study aimed to evaluate the clinical efficacy of levodopa–entacapone–carbidopa intestinal gel (LECIG) in 50 patients initiated during a four-year period at a tertiary movement disorders center. Motor outcomes were analyzed using Wilcoxon signed-rank and McNemar’s tests, while multivariable logistic regression was employed to identify predictors of improvement. **Results**: LECIG initiation significantly reduced mean daily OFF time from 4.63 ± 0.75 to 1.62 ± 1.97 h (*p* < 0.0001) and total dyskinesia duration by 65% (*p* < 0.0001). Furthermore, the prevalence of early morning akinesia decreased from 80% to 26%, and delayed ON phenomena were completely eliminated (*p* < 0.0001). Subgroup analyses indicated that patients with troublesome dyskinesia (≥1 h/day) achieved significantly greater reductions in involuntary movements compared to those with lower baseline dyskinesia levels (*p* = 0.013). **Conclusions**: These findings suggest that LECIG provides a meaningful and sustained stabilization of motor complications, highlighting its role as a valuable device-aided therapy in managing advanced Parkinson’s disease.

## 1. Introduction

Parkinson’s disease (PD) progresses—slowly or more rapidly in individual cases—to its advanced stage (aPD), characterized by profound motor fluctuations, dyskinesias, and debilitating non-motor symptoms that severely impair quality of life, with patients experiencing escalating disability and impaired activities of daily living (ADLs) [1,2], alongside fluctuating responses to standard oral therapies [1] and delayed gastric emptying [3,4], culminating in suboptimal symptom control.

Affecting over 50% of patients within 5–10 years of diagnosis, these complications arise from pulsatile dopaminergic stimulation, necessitating a paradigm shift toward continuous drug delivery to mimic physiologic neurotransmitter levels and stabilize motor function [5,6,7].

Device-aided therapies (DATs), such as levodopa–carbidopa intestinal gel (LCIG), levodopa–entacapone–carbidopa intestinal gel (LECIG), apomorphine subcutaneous infusion, foslevodopa–foscarbidopa subcutaneous infusion, and deep brain stimulation have emerged as transformative interventions for levodopa-responsive aPD patients with ≥5 daily doses, refractory OFF periods, and/or medication intolerance [5,8].

DATs fundamentally alter treatment trajectories by reducing OFF time by 2–4 h daily, minimizing dyskinesias, and enhancing “good quality ON” time without exacerbating troublesome involuntary movements. Unlike intermittent oral regimens, device-assisted infusions mitigate wearing-off phenomena through intrajejunal or subcutaneous infusion (via percutaneous endoscopic gastrojejunostomy (PEG-J) tubes or portable pumps), enabling individualized dosing (e.g., 16–18 waking hours) and the possibility of rapid adjustments with supplementary doses [9,10,11].

Long-term data confirm sustained efficacy: 4-year follow-ups demonstrate preserved motor scores, improved sleep, and quality-of-life gains, with high patient/physician global impression scores [9]. It must be noted however that these studies are usually on a small number of patients, providing limited experience.

LECIG, incorporating entacapone as a catechol-O-methyltransferase inhibitor alongside levodopa–carbidopa intestinal gel, exemplifies the evolution of device-assisted infusions. By extending levodopa bioavailability and inhibiting peripheral metabolism, LECIG achieves superior plasma stability versus standard LCIG, yielding greater OFF-time reductions (up to 4.5 h) and dyskinesia amelioration in real-world cohorts, alongside favorable tolerability (e.g., reduced nausea, neuropathy). Twelve-month observational studies report 85–90% continuation rates, with procedural complications (e.g., PEG-J dislodgement) managed via multidisciplinary oversight [10,12].

Current European tendencies expand DAT usage and redefine aPD management, bridging gaps in oral therapy limitations and underscoring the imperative for early specialist referral to optimize outcomes [5,6].

## 2. Materials and Methods

We retrospectively analyzed (pre-post analysis) standardized data from 50 consecutive patients initiated with levodopa–entacapone–carbidopa intestinal gel (LECIGON^®^, Britannia Pharmaceuticals Limited, Reading, UK) during a four-year period at our tertiary movement disorders center. Patient selection adhered to established aPD criteria defined by the Antonini et al. Delphi consensus [1], the CEPA study [13], and the 5-2-1 screening tool [14], corroborated by OBSERVE-PD findings [15], alongside Romanian national guidelines of the Romanian Neurological Society (https://neurology.ro/images/stories/uploads/protocoale/Ghid_SNR_Boala_Parkinson_final_3_decembrie_2024.pdf (accessed on 25 February 2026)). All aPD patients underwent multidisciplinary evaluation (movement disorder neurologist, psychiatrist, gastroenterologist, and psychologist) to determine optimal therapy, including available device-assisted options. As described previously [16], efficacy assessment incorporated a mandatory inpatient phase: 3–5 days of initial testing followed by 3–4 days of optimization of administration after PEG-J. Comprehensive documentation encompassed demographics, neurological status and fluctuations, prior regimens, adverse effects, and motor/non-motor complications. In all cases, we switched directly from conventional oral and transdermal treatment to LECIG therapy.

Statistical analysis was done using Prism 8.4.1 (GraphPad Software, San Diego, CA, USA). Paired pre/post-LECIG comparisons employed descriptive statistics and Wilcoxon signed-rank tests for continuous outcomes (means ± SD) and McNemar’s tests for binary prevalence. Intergroup differences (e.g., initial treatment subgroups, with/without dyskinesia subgroups) were assessed via Mann–Whitney U tests for non-parametric continuous data and Fisher’s exact tests for categorical variables. A secondary check using the Benjamini–Hochberg procedure to control the False Discovery Rate was performed and all results remained significant after this adjustment. Multivariable logistic regression was applied to evaluate predictors of improvement (age, gender, PD duration, and dopamine agonists/COMT inhibitor/MAO-B inhibitor usage), with results reported as odds ratios (ORs), 95% confidence intervals, and *p*-values. Significance was set at *p* < 0.05. Due to the observational nature of the study and the resulting imbalance in subgroup sizes, the subgroup comparisons and multivariable regression were considered exploratory. These analyses were intended to identify potential clinical trends rather than to provide definitive predictive evidence.

The study protocol was approved by the Ethics Committee of the George Emil Palade University of Medicine, Pharmacy, Science and Technology of Târgu Mureș (UMFSTGEP), Romania (approval number: UMFSTGEP 94/19 May 2017).

## 3. Results

### 3.1. Baseline Demographics and Clinical Characteristics

The study cohort consisted of 50 patients with advanced Parkinson’s disease (62% male, *n* = 31; 38% female, *n* = 19), exhibiting a mean age of 65.2 ± 8.65 years (range: 45–82 years; median: 67 years). Mean disease duration was 10.0 ± 4.01 years, with baseline cognitive function preserved (MMSE: 27.6 ± 1.8). Patients were on relatively high-dose levodopa regimens (mean: 857 ± 240 mg/day; range: 500–1750 mg), administered at 5.34 ± 0.56 doses daily. Adjunctive pharmacotherapy was prevalent: dopamine agonists (DA, 82%; pramipexole *n* = 10, 2.35 ± 0.87 mg; ropinirole *n* = 11, 12.36 ± 5.5 mg; rotigotine *n* = 21, 8.48 ± 1.89 mg), amantadine (28%, *n* = 14), apomorphine pen as rescue medication (14%, *n* = 7), MAO-B inhibitors (MAO-Bi, 60%, *n* = 30), and COMT inhibitors (COMTi, 70%, *n* = 35).

### 3.2. Primary Motor Fluctuation Outcomes Pre- and Post-LECIG

Initiation of levodopa–entacapone–carbidopa intestinal gel produced significant improvements in motor fluctuations (Table 1). Daily OFF time decreased from 4.63 ± 0.75 to 1.62 ± 1.97 h (*p* < 0.0001). Dyskinesia burden was markedly reduced: mild–moderate dyskinesia (prevalent in *n* = 33) was reduced from 2.65 ± 1.22 to 1.40 ± 0.56 h/day (*p* < 0.0001); severe dyskinesia resolved completely (*n* = 9; 2.78 ± 1.66 to 0 h, *p* = n.a.); and total dyskinesia was reduced from 3.41 ± 1.37 to 1.40 ± 0.56 h/day (*p* < 0.0001). Biphasic dyskinesia remained minimal (4% to 2%, *p* = 0.066). Secondary phenomena improved significantly: end-dose dystonia (30% to 4%, *p* = 0.0009), early morning akinesia (80% to 26%, *p* < 0.0001), delayed ON (44% to 0%, *p* < 0.0001), no ON (8% to 0%, *p* = 0.1175), sudden OFF (18% to 2%, *p* = 0.0157), and freezing of gait (56% to 28%, *p* = 0.0081). Hoehn–Yahr staging advanced in both ON (3.16 ± 0.37 to 3.04 ± 0.20, *p* = 0.0313) and OFF phases (4.18 ± 0.39 to 3.66 ± 0.48, *p* < 0.0001).

### 3.3. Impact of Baseline Treatment Regimens

Initial treatment heterogeneity (seven subgroups of treatment combinations—LD ± DA ± MAO-Bi ± COMTi, *n* = 5–19) yielded no significant baseline differences in motor parameters (all *p* > 0.05). Dichotomized analysis (levodopa with adjuncts but without DA vs. levodopa with adjuncts, including DA) revealed greater pre-switch biphasic dyskinesia in DA users (Mann–Whitney U = 24.5, *p* = 0.0008), with enhanced reductions of motor symptoms post-LECIG in patients without DA (*p* = 0.0008). Binary predictors (DA/COMTi/MAO-Bi presence) showed no significant effects except for the MAO-Bi trend for biphasic dyskinesia (*p* = 0.099). Multivariable logistic regression (age, gender, PD duration, and adjuncts, see Table 2) identified no significant predictors for mild–moderate dyskinesia improvement (all *p* > 0.05; PD duration *p* = 0.0746).

### 3.4. Subgroup Analysis of 5-2-1 Rule

Patients were categorized into two subgroups according to the 5-2-1 criteria: those fulfilling the dyskinesia threshold (≥1 h/day of troublesome dyskinesia, WD) versus those with <1 h/day dyskinesia (WoD), enabling targeted evaluation of treatment response heterogeneity (Table 3). WD patients (PD ≥ 5 years, OFF ≥ 2 h/day, dyskinesia ≥ 1 h/day; *n* = 41) versus WoD (*n* = 9) exhibited superior dyskinesia reductions: mild–moderate (median [IQR]: 1.00 [0.00–2.00] vs. 0.00 [−1.00–0.50] h; *p* = 0.0133, Mann–Whitney U); total dyskinesia (1.50 [0.00–2.00] vs. 2.50 [2.00–3.00] h; *p* = 0.0076). OFF reductions were equivalent (3.25 [3.00–3.50] vs. 3.00 [3.00–3.00] h; *p* = 0.1893). Secondary symptom improvement rates (dystonia, akinesia, ON phenomena, sudden OFF, and freezing) showed no intergroup differences (Fisher’s exact *p* > 0.05). Severe dyskinesia interpretation was precluded by baseline imbalance.

These preliminary observations suggest that treatment with LECIG is associated with a reduction in motor fluctuations across different patient profiles, with the most notable impact on troublesome dyskinesia appearing in patients with higher baseline burden. However, the imbalance in subgroup sizes limits the generalizability of these comparative findings.

## 4. Discussion

In Parkinson’s disease, the most convincing clinical improvement is observed following the administration of levodopa preparations. However, substitution therapy has several disadvantages; its unfavorable pharmacokinetics frequently triggers various motor complications (fluctuations, peak-dose and biphasic dyskinesias, dystonia, etc.), which represents a continuous challenge for clinicians (patients’ quality of life significantly deteriorates and therapeutic options become limited) [17,18,19]. Several pharmacological alternatives are widely used to mitigate these drawbacks (dopamine agonists, monoamine oxidase B inhibitors, and catechol-O-methyltransferase inhibitors). These options include the third-generation COMTi opicapone [20] and the MAO-Bi and glutamate modulator safinamide [21,22], which, however, have not yet been approved in Romania, nor in several other Central and Eastern-European countries [23].

In the advanced stages of Parkinson’s disease, continuously fluctuating motor complications can no longer be substantially improved with conventional oral and transdermal dopaminergic treatments. At this stage, DATs should be considered, with expected improvement of not only motor complications but also non-motor symptoms [24,25].

Continuous enteral infusion of levodopa makes it possible to bypass the gastroparesis, which is common in advanced Parkinson’s disease, thereby significantly improving the unfavorable pharmacokinetics of levodopa [4]. An additional advantage is that, if necessary, extra LD doses resulting in rapid clinical improvement can be administered in a personalized manner [11,26]. Recent studies have confirmed earlier observations that dyskinesias can be successfully improved with levodopa–carbidopa intestinal gel (LCIG) [27].

COMTis have been successfully used for nearly three decades to improve the unfavorable pharmacokinetics of standard LD formulations (they increase the half-life of levodopa, thereby improving its bioavailability). As a result, OFF periods can be effectively reduced in the long term [28]. The widely used entacapone is a potent, selective, peripherally acting COMTi that is administered at a fixed 200 mg dose with each levodopa dose [29]. Its pharmacokinetics, similarly to that of levodopa, is linear; its rapid absorption is not influenced by food intake (maximum plasma concentration can be reached within one hour) [30].

The combination of LCIG therapy with a COMTi (either entacapone and tolcapone) has been investigated previously. As a consequence of the combination, the LCIG dose could be reduced by 20% while maintaining stable plasma concentrations and adequate control of motor complications [31]. Levodopa–entacapone–carbidopa intestinal gel is one of the newest DAT alternatives. The formulation contains 20 mg/mL entacapone in addition to 20 mg/mL levodopa and 5 mg/mL carbidopa. As a result of COMT inhibition, the bioavailability of levodopa in the gel can be improved by 20–25% compared to LCIG, thus ensuring similarly stable and clinically effective plasma levodopa concentrations at a lower daily levodopa dose. In a randomized, open-label, crossover study, the clinical efficacy of LECIG was found to be comparable to that of LCIG [32]. Patient selection and the initiation procedure are performed similarly to LCIG therapy, via percutaneous endoscopic gastrostomy [33]. The first clinical experience was reported in a 2021 publication; however, real-world clinical data from everyday practice are still limited [34,35].

The multidisciplinary team at our clinic has more than fifteen years of experience with intrajejunal levodopa administration [36,37]. In our previous publications, we have analyzed in detail the comprehensive assessment of our patients for suitability for device-aided therapies, the specific characteristics and limitations of the applied dopaminergic replacement therapy, as well as the spectrum of motor and non-motor complications [37,38,39,40]. We have demonstrated the long-term efficacy and safety of LCIG therapy even in our patients with severe, complex dyskinesias [16], a benefit also described in a recently published post hoc analysis [41].

The present findings suggest a favorable clinical response of levodopa–entacapone–carbidopa intestinal gel in mitigating motor fluctuations among 50 patients with aPD, characterized by prolonged OFF periods, disabling dyskinesias, and advanced Hoehn–Yahr staging. The observed 3.01 h significant reduction in OFF time, the near-complete resolution of severe dyskinesia, and a 65% decrement in total dyskinesia duration align with the previously described [5] steady-state pharmacokinetics of continuous intrajejunal infusion, which circumvents pulsatile levodopa delivery inherent to oral regimens. The improvements observed in our study align with established thresholds for clinical meaningfulness. Our findings are consistent with the 12-month ELEGANCE study, which reported a reduction in OFF-time exceeding 3 h alongside improved MDS-UPDRS Part IV scores and quality-of-life metrics [12]. Furthermore, the 71% reduction in early morning akinesia and the resolution of delayed or ‘no ON’ phenomena noted in our cohort underscore the clinical utility of LECIG in addressing complex motor fluctuations. Although there is a penury of data regarding the direct comparison of LCIG and LECIG, recent studies showed a favorable effect of entacapone-mediated COMT inhibition, which extends levodopa bioavailability and stabilizes plasma levels despite a 20% dose reduction [32].

Subgroup analyses revealed nuanced predictors of response. Patients with troublesome dyskinesia (≥1 h/day) exhibited amplified dyskinesia amelioration, suggesting this triad optimally identifies LECIG beneficiaries, consistent with eligibility guidelines. Baseline regimen heterogeneity yielded no prognostic disparities except borderline biphasic dyskinesia modulation, with levodopa monotherapy patients showing enhanced post-switch gains; multivariable modeling confirmed PD duration as a near-significant covariate. These observations extend prior real-world evidence, where LECIG efficacy persistence exceeded LCIG at 365 days despite higher costs, with sustained Hoehn–Yahr improvements, mirroring our ON/OFF shifts [8,42,43]. Our results are corroborated by existing literature across diverse geographical and socioeconomic cohorts, which consistently demonstrates the therapeutic efficacy of LECIG treatment. This alignment with international data underscores the generalizability of our findings across varying patient demographics [35,43,44].

Several studies also showed that the procedure is safe; serious complications are rare and less than 10% of patients stop treatment due to dissatisfaction [43,44,45]. Our data seem to support this: no perioperative complications or immediate device-related adverse events were reported during the initiation of LECIG therapy. In a subset of patients who already had at least 18 months of follow-up, the treatment demonstrated a favorable safety profile with no therapy discontinuations. Adverse events were limited to six non-severe occurrences (one case of sialorrhea, two patients with weight loss, two patients experiencing polyneuropathy, and one patient with depression) and one unrelated death due to cardiac arrest.

A notable strength of this investigation is the inclusion of 50 patients, representing a comparatively large sample size for real-world LECIG studies conducted in tertiary settings. Unlike smaller observational series (typically 20–35) our study population consists of a sizable group of subjects with complex, multidrug-refractory aPD patients (mean PD duration over 10 years; high-dose levodopa usage). While the numerical imbalance between subgroups (e.g., 5-2-1 rule: WD *n* = 41 vs. WoD *n* = 9) necessitates a cautious interpretation, this cohort size allows for a comprehensive descriptive evaluation of treatment response in a specialized environment. These findings suggest that our results, from a single tertiary center, are applicable to similar expert-led environments, where multidisciplinary management optimizes outcomes and manages procedural risks [12,39].

As demonstrated in our previous publications, the treatment of Parkinson’s disease, in our tertiary center and also at the national level, is carried out in accordance with international guidelines, naturally adapted to local conditions, therapy availability and the individual characteristics of each patient [46,47,48]. Considering that the available treatment options in Romania are broadly similar to those in other Central and Eastern-European countries, we believe that this type of analysis may assist practicing neurologists in assessing more advanced stages of PD, as well as in the earlier identification of patients for whom jejunal levodopa infusion may result in a significant improvement in quality of life [49].

This study harbors several limitations inherent to its observational, single-arm design. Foremost, the absence of a randomized control arm precludes definitive causal attribution of LECIG’s effects, potentially confounding spontaneous disease stabilization or placebo responses. The single-center tertiary recruitment, while yielding a sizable cohort, introduces selection bias toward multidrug-refractory patients, limiting generalizability to community or early aPD settings. Short-term follow-up (pre/post-switch only) omits longitudinal attrition, non-motor outcomes, and safety endpoints (e.g., PEG-J complications, neuropathy incidence), which are critical given the 15–20% 5-year discontinuation rates described in the literature [6,9,37]. Also, reliance on retrospective chart data risks incomplete motor fluctuation logging. Furthermore, the subgroup analyses suffered from marked imbalance and small group sizes, which likely compromised statistical robustness and inflated Type II error. Similarly, the multivariable regression analysis is underpowered due to the modest sample size, despite the presence of several near-significant trends. Consequently, these specific findings should be viewed as hypothesis-generating rather than definitive, requiring validation in larger, multi-center prospective cohorts. Finally, the homogenous cohort (MMSE 27.6 ± 1.8; 62% male) possibly underrepresents dementia, frailty, or diverse demographics, necessitating multicenter RCTs for broader validation.

## 5. Conclusions

In context, LECIG represents a pivotal DAT evolution, bridging oral therapy gaps by enhancing “good ON” time without exacerbating troublesome dyskinesias. Compared to LCIG, entacapone integration yields superior OFF reductions (up to 4.5 h) and tolerability, positioning it as first-line for COMTi-eligible patients per emerging European approvals. Future randomized trials should validate symptom stratification, incorporate biomarkers, and evaluate transitions to gene therapies amid inexorable progression. These data affirm early specialist referral to optimize DAT trajectories in aPD.

## Figures and Tables

**Table 1:** Primary motor fluctuation change, pre- and post-LECIG (*N* = 50).

Motor Parameter	Pre-LECIG	Post-LECIG	*p*-Value
OFF duration (h/day)	4.63 ± 0.75	1.62 ± 1.97	<0.0001
Mild–moderate dyskinesia (h/day, *n* = 33→29)	2.65 ± 1.22	1.40 ± 0.56	<0.0001
Severe dyskinesia (h/day, *n* = 9→0)	2.78 ± 1.66	0.00	n.a.
Total dyskinesia (h/day)	3.41 ± 1.37	1.40 ± 0.56	<0.0001
Hoehn–Yahr score ON	3.16 ± 0.37	3.04 ± 0.20	*p* = 0.0313
Hoehn–Yahr score OFF	4.18 ± 0.39	3.66 ± 0.48	<0.0001
End-dose dystonia (% of patients)	30.0	4.0	0.0009
Early morning akinesia (% of patients)	80.0	26.0	<0.0001
Delayed ON (% of patients)	44.0	0.0	<0.0001
Sudden OFF (% of patients)	18.0	2.0	0.0157
Freezing of gait (% of patients)	56.0	28.0	0.0081

**Table 2:** Multivariable logistic regression for important predictors for dyskinesia improvement.

Predictor	OR	95% CI	*p*-Value
Age	0.963	0.894–1.038	0.326
PD duration	1.224	0.980–1.529	0.075
MAO-Bi present	1.152	0.343–3.878	0.819

**Table 3:** Analysis of subgroups with more than 1 h/day of troublesome dyskinesia and without.

Outcome (Median [IQR] Reduction, h)	WD (*n* = 41)	WoD (*n* = 9)	*p*-Value
Mild–moderate dyskinesia	1.00 [0.00–2.00]	0.00 [−1.00–0.50]	0.013
Total dyskinesia	1.50 [0.00–2.00]	2.50 [2.00–3.00]	0.008
OFF duration	3.25 [3.00–3.50]	3.00 [3.00–3.00]	0.189

## Data Availability

The original contributions presented in this study are included in the article. Further inquiries can be directed to the corresponding author.

## Abbreviations

The following abbreviations are used in this manuscript:

ADL	activities of daily living
aPD	advanced Parkinson’s disease
COMTi	COMT inhibitor
DA	dopamine agonist
LCIG	levodopa–carbidopa intestinal gel
LD	levodopa
LECIG	levodopa–entacapone–carbidopa intestinal gel
MAO-Bi	MAO-B inhibitor
PD	Parkinson’s disease
PEG-J	percutaneous endoscopic gastrojejunostomy
WD	with more than 1 h/day dyskinesia
WoD	less than 1 h dyskinesia
